# Agro-Fruit-Forest Systems Based on Argan Tree in Morocco: A Review of Recent Results

**DOI:** 10.3389/fpls.2021.783615

**Published:** 2022-01-05

**Authors:** Abdelghani Chakhchar, Imane Ben Salah, Youssef El Kharrassi, Abdelkarim Filali-Maltouf, Cherkaoui El Modafar, Mouna Lamaoui

**Affiliations:** ^1^Laboratoire de Biotechnologie et Physiologie Végétales, Centre de Biotechnologie Végétale et Microbienne Biodiversité et Environnement, Faculté Des Sciences, Université Mohammed V de Rabat, Rabat, Morocco; ^2^Laboratory of Biotechnology and Molecular Bioengineering, Department of Biology, Faculty of Sciences and Techniques Guéliz, Cadi Ayyad University, Marrakech, Morocco; ^3^African Sustainable Agriculture Research Institute (ASARI), Mohammed VI Polytechnic University (UM6P), Laâyoune, Morocco; ^4^Laboratory of Microbiology and Molecular Biology, Department of Biology, Faculty of Sciences, Université Mohammed-V de Rabat, Rabat, Morocco; ^5^Laboratory of Developmental Biology of Plants, Institute of Botany, Justus Liebig University Giessen, Giessen, Germany

**Keywords:** argan tree, ecophysiology, arganiculture, conservation, domestication, argan oil

## Abstract

The argan tree, *Argania spinosa* (L.) Skeels, is a horticultural forestry species characterized by its endemicity and adaptation to arid and semi-arid zones in the southwest of Morocco. Despite its limited geographical distribution, argan tree presents large genetic diversity, suggesting that improvement of argan is possible. This species plays important ecological, and socioeconomic roles in the sustainable development of the country. The integration of arganiculture into Moroccan agricultural policy has been implemented through a sector strategy, which is fully aligned with the conservation and regeneration of argan forest. *A. spinosa* is suitable for incorporation into different agroforestry productive systems under agro-fruit-forest model and its domestication will provide a powerful means of socio-economic and environmental management. Here, we provide an overview of the argan tree literature and highlight the specific aspects of argan stands, as agro-forest systems, with the aim of developing an adequate strategy of conservation and domestication of this species. We introduce promising programs and projects for argan plantations and arganiculture, which have been adopted to relieve anthropogenic pressure on the natural argan forest.

## Introduction

The argan tree, *Argania spinosa* (L.) Skeels, is a horticultural forestry species endemic to Morocco that has multiple uses. It plays an important role, mainly for the local population, in terms of its botanical, ecological, and economic interest as well as its social value (Benabid, 1985; M’Hirit et al., 1998; Msanda et al., 2005; Lefhaili, 2010). The main interest is focused on its fruit, which produces very valuable oil that is not only used for food but also for cosmetic and medicinal purposes (Lybbert et al., 2010). The habitat of this species is spread over important areas of the natural forests in North Africa, especially in southwestern Morocco. The argan tree contributes to the preservation of the ecosystem and provides an environment conducive to maintaining floristic and faunistic biodiversity. *A. spinosa* forms a forest area called the “argan region,” covering an area of 3,976,000 ha, spanning from the city of Safi in northeastern Morocco to the Saharan fringe in the south, where the argan tree occupies about 70% of the woodland area (M’Hirit et al., 1998). The past and present regression of argan forests, in terms of both density and total area, is mainly due to desertification, population pressure, pastoral activities, and the overexploitation of forest resources by the local population (McGregor et al., 2009; de Waroux and Lambin, 2012; El Wahidi et al., 2014; Karmaoui, 2016; Genin et al., 2017). Furthermore, this natural forest is expected to be greatly threatened by the effect of global climate change in the future. The climatic conditions of southwestern Morocco are highly arid. Due to its geographic location at the frontier of one of the hottest deserts in the world, as well as the predicted impacts of climate change on the area—with an estimated temperature increase of 0.5–1°C by 2020 and 1–1.5°C by 2050 and 2080 [Convention-Cadre des Nations Unies sur les Changements Climatiques [CCNUCC], 2016] and an overall decrease in annual rainfall of 5–15% in the southern provinces by 2050 (Terink et al., 2013) the natural argan forest is expected to face major challenges. In fact, drought is causing significant reductions in the natural distribution of argan trees, with the disappearance of the most exposed and vulnerable trees. This situation has been aggravated by the low rate of soil recovery due to the use of inappropriate farming methods and overgrazing (McGregor et al., 2009; de Waroux and Lambin, 2012; Genin et al., 2017).

Like many seed plants, the argan tree is naturally propagated by seeds. Its fructification usually begins at the age of 5 years, and the fruit yield depends on the genotype, age of the tree, management practices, and other factors, including climate and soil conditions. The argan species display a wide genetic diversity, which is evident even within the same locality and under similar ecogeographical conditions. This diversity provides a broad genetic basis for domestication and breeding programs (Mouhaddab et al., 2015, 2016b, 2017; Yatrib et al., 2015; Pakhrou et al., 2016; Chakhchar et al., 2017a). The reproductive mode of *A. spinosa*, which is essentially allogamic, may explain the origin of such diversity.

Given the importance of this species, the development of sustainable strategies for the preservation, production, and propagation of argan trees is necessary. The preservation of the natural argan forest consisted in developing congestion between the different stakeholders in its range. The involvement of the local beneficiary population in this strategy with the protection of the biosphere and the soil against erosion will be the main keys to promote the sustainable development of this species. By limiting the access for a predetermined time to the harvest of the argan fruits from the natural forest allows its natural regeneration. On the other hand, the rehabilitation of degraded areas in their natural range by planting argan trees and its extension next to this range as well as the promotion of argan nurseries will greatly increase the production of trees and fruits and improve the oil yield. The domestication of the argan tree and its cultivation in modern orchards would turn it into an oil seed crop for oil production, which opens up important economic opportunities for Morocco in the face of the growing demand for quality argan oil and will help reduce the pressure on the wild argan forest.

Scientific research and biotechnology tools can offer improved management methods to rehabilitate soil-degraded areas as well as expand the cultivation area to regions previously unsuitable for cultivation. Selection of healthy cultivars with good genetic and agronomic qualities, the use of modern vegetative propagation techniques and the implementation of good agricultural practices will allow a controlled process of argan cultivation, as a viable tree crop, and monitoring its environment. These scientific research assets will improve protection, production and domestication strategies by ensuring good quality and quantity argan production and increasing the value of the supply chain of its products.

This review is organized as follows: Section 1 gives a general overview on botanical and phenological description of argan tree and its cytological, genetic and growth aspects. Section 2 provides an insight on the biogeographical distribution of *A. spinosa* and its edapho-climatic requirements and discusses its ecophysiology. Section 3 highlights the ecological and socio-economic interests of the argan tree. The last section deals with the regeneration and domestication strategies of the argan tree and the implementation of the arganiculture strategy as a profitable horticultural industry.

## Botanical, Genetic And, Biotechnological Aspects

### Botanical, Phenological, and Taxonomical Aspects

**FIGURE 1:**
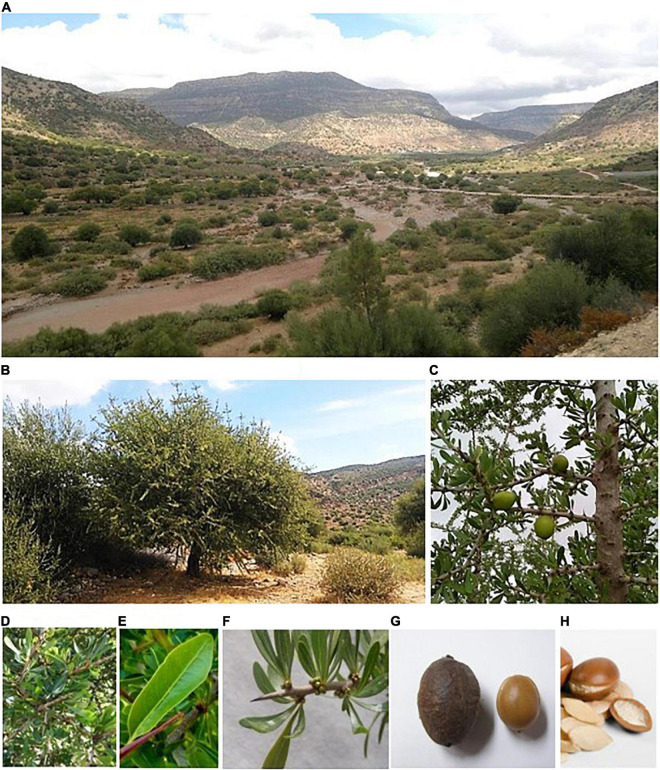
Different aspects and parts of *Argania spinosa* **(A)** argan forest, **(B)** adult argan tree, **(C)** argan branch bearing immature fruits, **(D,E)** argan leaves, **(F)** argan nodal stem, **(G)** fruit and seed of argan tree, and **(H)** argan nuts.

The argan tree belongs to the Sapotaceae family (Ericales order), which includes over 50 genera and 800 species. Sideroxyleae is a Sapotaceae subfamily of about 80 species of trees and includes *Argania spinosa* as the only species of the genus *Argania* (Pennington, 1991). The argan tree can reach 6–8 m in height and resembles an olive tree (Figure 1). Its trunk is knotty, often formed of several intertwined stems, and branching begins about 1 m from the ground, resulting in great morphological diversity (Zahidi et al., 2014b). Its foliage is semi-persistent and can fall during a severe drought. The leaves are alternate, coriaceous, entire, lanceolate, or spatulate, attenuated at the base in a short petiole, and often united in fascicles. They have a very distinct median vein and they are very thin and branched lateral veins.

*Argania spinosa* is a monoecious species whose flowers are hermaphrodites grouped into glomeruli (15 flowers or more) or are found as solitary flowers (Bani-Aameur, 2000). Flowering begins with the first rains of December and continues until March and April, but they can also appear in any season. Spring is, however, the most favorable period. The flowers are tiny (2–4 mm in diameter) and usually appear at the base of the leaves or on the nodes of the mature branches in axillary position on the twigs (Bani-Aameur, 2000). Pollination is mainly entomophilous, rather than anemophilous (Nerd et al., 1998; Mouhaddab et al., 2016b). Under natural conditions, pollen fertility varies from 49.1 to 100%, with significant variability between trees (Bani-Aameur, 2002).

The fruits generally ripen between June and August, depending on the location and environmental conditions. The fruits of the argan tree exhibit various phenotypes (Bani-Aameur et al., 1999; Bani-Aameur and Ferradous, 2001; Ait Aabd et al., 2011, 2012; Zhar et al., 2016), being false drupes (according to Rammal et al., 2009) of size ranging from olive to walnut with different morphotypes (oval, rounded, and fusiform). The fruit of *A. spinosa* is very special; there is a hard stone (seed) under the fleshy pulp with 2–3 (or even 4 or 5) cavities containing false almonds (nuts). At maturity, the fruit color evolves toward yellow or red. Some argan trees produce fruit every year, others every other year, and others with a 3-year periodicity. There may be one to three seeds per fruit, often only one, the others having aborted. They have an oval shape, a brown color, a smooth aspect with an average length of about 2 cm (Figure 1G). The test of the argan seed is composed of very thick sclerotized cells conferring to this seed an exceptional hardness. The seed contains whitish nuts (very bitter and rich in oil) surrounded by a very thin, tangled network of loose, woody, vascular strands tracing wrinkles on its outer surface (Morton and Voss, 1987).

Argan trees have taproots that can plunge deeply into the ground, which allows for the recovery of water from up to about 8 m depth (Ain-Lhout et al., 2016), and an adventitious root system that traps water in the surface soil (Chakhchar et al., 2018a). This root system benefits from an important mycorrhizal symbiosis (Nouaim and Chaussod, 1994; Nouaim et al., 1994).

### Cytological and Genetic Aspects

The argan tree is a predominantly outcrossing species, which may contribute to its genetic diversity and resilience. However, the cytology of the argan tree is poorly understood. The karyotype of *A. spinosa* consists of ten small pairs of chromosomes (2n = 2x = 20), which is the lowest number within the Sapotaceae. The proposed putative karyotype is represented by six pairs of metacentric and four pairs of submetacentric chromosomes (Majourhat et al., 2007); however, Johnson (1991) has suggested that the number of basic chromosomes in Sideroxyleae is *x* = 11, while the most common chromosome number of species in the Sapotaceae family varies between *x* = 12, 13, and 14 (Bawa, 1973; Ono, 1977; Johnson, 1991).

The evaluation of the argan genetic variations within and amongst its natural population in Morocco was widely studied along with its genetic structure covering the whole argan distribution area. Those studies were meant in particular for the conservation of the argan genetic resources and their prevention from extinction. The identification of superior planting material to use as a genetic basis for the future breeding programs, the forest regeneration, the domestication under arid and semi-arid environments and finally for the estimation of population’s differentiation and how the genetic patterns are linked to the geographical distributions.

The investigation of the diversity was done through classic morphological and biochemical markers (El Mousadik and Petit, 1996b; Bani-Aameur, 2004; Ait Aabd et al., 2011, 2012; Zahidi et al., 2014a; Metougui et al., 2017; Chakhchar et al., 2018b) revealing a high genetic variability. From a conservation perspective, more phenotypic, biochemical, and physiological parameters were as well studied in order to make a primary trees selection, assess provenance variations and estimate population differentiation based in particular on oil yield, seed’s crushing abilities and to drought adaptation (Ait Aabd et al., 2010; Chakhchar et al., 2017a, b). The characterization of the genetic variability was evaluated as well through molecular approaches, confirming the important genetic diversity among the argan population. Different types of DNA markers were successfully applied such as RAPD Markers (Majourhat et al., 2008), AFLP markers (Pakhrou et al., 2016), SSR markers (Majourhat et al., 2008; El Bahloul et al., 2014; Chakhchar et al., 2017a), ISSR markers (Ait Aabd et al., 2015; Mouhaddab et al., 2015, 2016a; Yatrib et al., 2015; Pakhrou et al., 2017), and IRAP (Pakhrou et al., 2017). The results of these studies show with consistency that argan genetic diversity is high. The polymorphism was assessed also in the maternally inherited markers cpDNA, to assess the genetic structure of populations, revealing the predominance of seed-mediated over pollen-mediated gene flow in argan tree (El Mousadik and Petit, 1996a). The assessment of the allelic richness was as well considered to point out the potential individuals prior to conservation (El Mousadik and Petit, 1996b; Petit et al., 1998).

More recent work has shown that, despite the limited geographical distribution of the argan tree in southwestern Morocco, variation between ecotypes collected from different provenances occurs (El Bahloul et al., 2014; Chakhchar et al., 2017a). Chakhchar et al. (2017a) reported that the majority of genetic variation within four argan provenances (71%) is recorded between individuals. This raises the possibility that phenotypic screening and DNA/RNA analysis could be used to identify and develop traits/varieties that confer further tolerance to various stresses and enable the maintenance of argan production, as climate change is expected to result in more frequent and severe weather events in Northern Africa. Population genetic analyses using microsatellite markers have revealed a high degree of relatedness between contrasting *A. spinosa* provenances, indicative of both artificial selection and the transport of ecotypes between different provenances throughout centuries of management of the argan forest (Chakhchar et al., 2017a).

Therefore, all the available data generated regarding the genetic diversity and allelic richness in *A. spinosa* based on molecular marker analyses may be used to preserve the genetic argan tree resources, preconize a natural reserve concept, and improve the genetic richness of this species, which should be a priority for the Moroccan argan forest. However, many gaps remain to be filled in terms of research in this area. Further inputs are required regarding the genomic basis of argan to improve our knowledge of the bottlenecks that limit argan domestication and breeding. Recently, a draft genome of *A. spinosa* assembly was generated by a hybrid *de novo* assembly method that combines short- and long-sequencing reads (Khayi et al., 2018).

### Germination, Growth, and Multiplication

Due to heavy animal and human pressure and past climate change scenarios, the argan forest has undergone a severe decline (Alados and El Aich, 2008; Díaz-Barradas et al., 2010; Defaa et al., 2013). Moreover, adverse climatic and environmental factors have prevented its natural regeneration (Bani-Aameur and Ferradous, 2001). Measures for the natural resources conservation and rejuvenation are reported to be crucial. Therefore, multiplication methods are urgently required to meet the growing need for the conservation and propagation of this multipurpose tree.

Presently, the only possible way to multiply argan in Morocco is through seedling (Defaa et al., 2011; Ferradous and Hafidi, 2018). On average, only 30% of *A. spinosa* seeds germinate due to the combined effect of non-viability and dormancy. The seeds germination is strongly genotype-dependent which limits the natural turnover of *A. spinosa* in native conditions (Bani-Aameur and Alouani, 1999). Further, its germination is affected by salt and drought stress (Bouzoubaâ and El Mousadik, 2003). However, the germination efficiency can be improved and germination rates of over 80% were reached through diverse practices such as high-performing genotypes selection, seeds conservation under cold conditions, sterilization, scarification, light pre-treatment, temperature pre-treatment, soaking in water, pre-treatment with some germination promoters (gibberellic acid and potassium nitrate), or even through *in vitro* techniques (Nouaim et al., 2002; Alouani and Bani-Aameur, 2003, 2004; Al-Menaie et al., 2007; Justamante et al., 2017).

However, with the lack of well-established argan standard varieties, the seedlings are not the best option for oil production as they can be genotypically different from the parental trees due to the high heterozygosity of the tree. The random genetic mixing may result in poor, irregular productivity and deficiency in superior trees with an important impairment in oil production. Thus, the use of seedling will fit more adequately an environmental and ecological approach rather than an oil industry approach.

The possibility to produce genetically identical plants from selected superior genotypes would significantly increase the argan tree profitability and certify the transmission of superior traits to the offspring plantations. The shift from the sexual seed propagation to vegetative propagation is highly recommended to ensure the switch from being a wild tree to an oil crop (Bonga, 2016). The argan tree clonal propagation was done only through nursery techniques (Bellefontaine et al., 2010; Ferradous et al., 2011). Bud culture was also considered, however, it was revealed to be challenging to establish with success due to many problems, especially the mature age related problems (Bousselmame et al., 2001; Nouaim et al., 2002).

Moreover, attempts to multiply argan through cuttings, grafting, and marcotting (air-layering) were already realized; only the rooting process poses difficulties. For vegetative propagation, lignified stem cuttings collected from adult trees were originally used, while softwood cuttings grown under axenic conditions became subsequently used (Nouaim et al., 2002). Grafting as well as marcotting, were established as alternative techniques to mobilize and rejuvenate older argan trees (Bellefontaine, 2010; Bellefontaine et al., 2010). However, these methods are time- and labor-intensive and limited by low rooting rates (Bousselmame et al., 2001; Nouaim et al., 2002; Lamaoui, 2015).

As the number of population genetic studies on argan trees increases, the characterization and identification of its effective population size and gene flow will allow for the development of a future argan tree breeding program by ensuring that domesticated populations have sufficient genetic diversity. Thus, the clonal propagation of mature elite-trees of *A. spinosa* through innovative tissue culture systems, such as the direct somatic embryogenesis technique, for the artificial seed production could be an alternative to meet the growing need for the clonal multiplication of potentially high-production individuals. Somatic embryogenesis could be an alternative technique and is one of the most promising approaches by means of which haploid or somatic bipolar cells are developed through different embryogenic stages, giving rise to a complete plant. Once mastered, this technique may make clonal production possible for an increasing number of elite argan plants in a much-reduced physical area, for the propagation and preservation of argan biodiversity.

## Biogeographical Range and Ecophysiology

### Biogeographical Distribution

**FIGURE 2:**
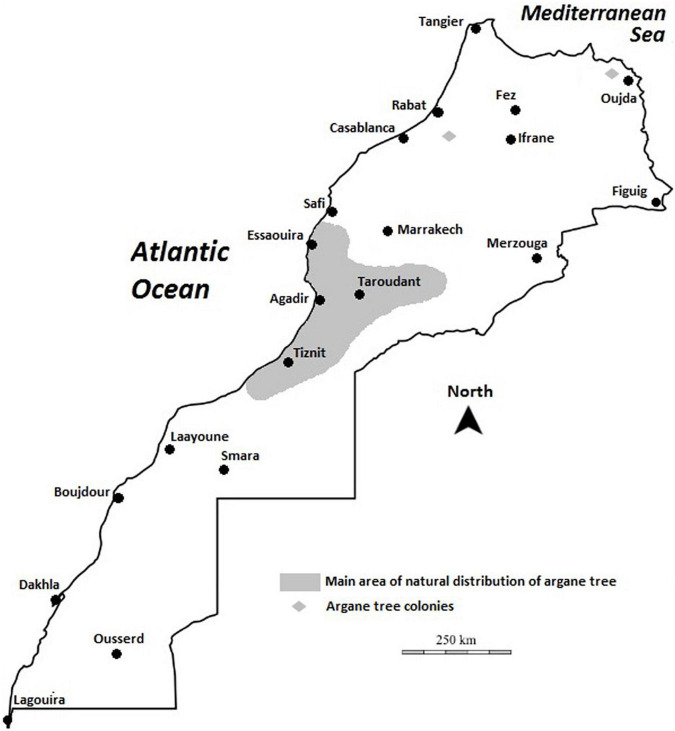
Map of Morocco showing the biogeographical distribution of the argan tree.

Argan forests cover the largest forest area in Morocco, after holm oak (*Quercus ilex* L.) and Cedar (*Cedrus atlantica* Manetti ex Endl.). The argan forest, which forms vast natural areas (also called “arganeraie”), is currently estimated to cover an area of 800,000 (Msanda et al., 2005) to 950,000 ha (Lefhaili, 2010) and holds more than 20 million trees. The main area of its distribution is limited to the southwest of the country, in the Agadir region, north of Oued Draa and south of Oued Tensift (Msanda et al., 2005) between 29° and 32° north (Figure 2). The argan tree is the northernmost species of the tropical Sapotaceae family. It grows in vast and geographically diverse territory, including coastal and inland sectors, plains, and mountainous areas. In addition, argan is found in some outlying areas, near Rabat in the northeast and in the Beni-Snassen mountains in the northwest of Morocco, attesting to its previous range of expansion; see Figure 2. The current area of *A. spinosa* encompasses the entire Souss watershed (Taliouine–Aoulouz–Taroudannt–Agadir), the southern and western foothills of the Western High Atlas, the plateaus of Haha and Ida-ou-Tanane region, and from the mountains of the southwest Anti-Atlas to Sidi Ifni in the southeast (Tarrier and Benzyane, 2003; Msanda et al., 2005). Prospection carried out in northeastern Morocco has assembled a synthetic map of the geographical area of argan, indicating its current state in the western Beni-Snassen foothills and its existence in the eastern Rif on the plain of Bou-Areg (Tazi et al., 2003; Faouzi et al., 2014).

The argan ecosystem is divided into two quite distinct habitats: (i) the plain of argan orchards (less dense forest), which has an average density of 10 trees/hectare, and (ii) the mountain argan forest (tendency to clear forest), a somewhat original model, with a density that can reach 500 trees per hectare. The argan forest is confined to the non-cultivable parts of the maritime coastline and rugged mountain areas (M’Hirit et al., 1998; Msanda et al., 2005).

### Edapho-Climatic Requirements

The argan tree grows naturally in arid and semi-arid regions in southwestern Morocco. In this Mediterranean ecosystem, the climate is arid, and rainfalls unevenly distributed over the year. The habitat of argan trees is part of the Mediterranean–Saharan transition zone (McGregor et al., 2009). *A. spinosa* is a thermophilic and xerophilic tree of hot and temperate arid climate (along the coast and in the plains), warm semi-arid and temperate (flanks of the High Atlas and Anti-Atlas), or even Saharan further south (Msanda et al., 2005). It is well-adapted to growth under conditions characterized by low rainfall and temperatures rising above 40°C. The annual precipitation in its main distribution area is between 150 and 400 mm. Further south, along temporary streams in desert areas (annual rainfall <100 mm), adult trees can survive under very dry conditions for long periods by accessing deep water sources or using water runoff (Msanda et al., 2005). Thus, argan may serve as a crop in desert areas (Nerd et al., 1994); however, the argan tree is not well-adapted to salinity (Bani-Aameur and Sipple-Michmerhuizen, 2001; Bouzoubaâ and El Mousadik, 2003). Furthermore, low temperatures limit its extent, in terms of altitude. However, it can grow on steep slopes of the Western High Atlas and Anti-Atlas, from sea level to 1,500 m (Msanda et al., 2005).

Oceanic atmospheric humidity appears to be an essential factor in the distribution of the species, partially compensating for drought conditions in the argan ecosystem (Msanda et al., 2005). These oceanic influences contribute to the increase in relative humidity (RH) which reduces evaporation demand by reducing the vapor pressure deficit (VPD) gradient between the interior of the leaves and the surrounding air. A low VPD promotes the opening of stomata and facilitates the inflow of CO_2_ into the leaf mesophyll, which can improve photosynthesis with relatively low water loss (Oksanen et al., 2018). Summer cloudiness, accompanied by a very high relative humidity (which frequently exceeds 90% during many months of the year, especially in summer and autumn), constitutes one of the major climatic features of the geographical area of the argan tree. Fogs and dews, often associated with low cloud formations, are the cause of significant nightly precipitation (Msanda et al., 2005).

Argan can adapt to all soil types, except moving sands. In the natural forest, most argan trees grow on shallow, rocky, and poor soils, allowing its growth on shales, quartzites, limestone, or alluvium (M’Hirit et al., 1998; Nouaim, 2005). Like some plant species, *A. spinosa* has a dimorphic root system that appears to be linked to a flexible water absorption model (Chakhchar et al., 2018a). Due to its root system, this tree can be found on poor and shallow soils. Its taproots continue the proliferation in depth and the roots that develop horizontally, allowing the absorption of nutrients near the surface and the absorption of water from shallow to deep soil layers when the surface dries (Chakhchar et al., 2018a).

### Ecophysiology

The argan tree is located in a restricted area in southwestern Morocco, characterized by low water availability and high evapotranspirative demand. Despite the adaptation of the argan tree to drought stress, the extent of native argan forest has decreased significantly as a result of increased aridity, land-use changes, and the expansion of olive cultivation (de Waroux and Lambin, 2012; Alba-Sánchez et al., 2015). A study of germination and seedling survival under experimental saline conditions showed that argan tree did not behave to salinity as a halophyte but rather as a salt-sensitive glycophyte (Bani-Aameur and Sipple-Michmerhuizen, 2001). However, the argan tree can withstand drought by adopting mechanisms that limit or slow down the reduction of water potential, which are generally part of a well-adapted water conservation strategy. It has developed the capacity to trap atmospheric moisture, to then directly use it, and also to store water in the roots for use in dry conditions (El Aboudi et al., 1991; Peltier et al., 1992; Msanda et al., 2005). The ecophysiological traits contributing to the efficient water use of argan are loss of leaves, flowers, and fruits under drought conditions; the exploitation of deeper soil horizons during the dry season due to the length of its woody roots (about 8 m); the water withdrawal from tree reservoirs such as trunk stems and branches; and the diurnal closure of the stomata, which are regulated by several stimuli (El Aboudi et al., 1991; Peltier et al., 1992; Ferradous et al., 1996; Msanda et al., 2005; Ain-Lhout et al., 2016). This tree has a very efficient water transport system to exploit the available soil moisture (Ain-Lhout et al., 2016), which also allows it to grow at large distances from the ground water. The water transport system in the argan tree is characterized by its high hydraulic conductivity of the xylem, and by the maintenance of water absorption through increased rooting (Chakhchar et al., 2018a). In addition, during severe drought, argan tree reduces water losses from the foliage by increasing the epicuticular wax load (Chakhchar et al., 2015b), controlling stomatal conductance and transpiration (Chakhchar et al., 2015c, 2017a) and decreasing the total foliar surface (Díaz-Barradas et al., 2010, 2013; Chakhchar et al., 2018b). *A. spinosa* ecotypes collected from coastal, inland, and mountainous Moroccan regions showed differential physiological, morphological, and phenological responses to seasonal variations in temperature, relative humidity, and water availability (Bani-Aameur and Zahidi, 2003; Bouzoubaâ et al., 2005; Díaz-Barradas et al., 2010; Zahidi et al., 2013a, b,c). A close coordination of stomatal conductance (gs) with leaf-air vapor pressure deficit and the leaf water potential has been recorded in *A. spinosa* under natural and experimental conditions (Díaz-Barradas et al., 2010, 2013; Chakhchar et al., 2015b, c, 2016, 2018b). Closure of argan stomata under low moisture could be the result of a decrease in bulk foliar water potential as leaf-air VPD increases (Díaz-Barradas et al., 2010, 2013). The effect of low humidity (for example, under drought conditions) on tree argan stomata is consistent with a decrease in CO_2_ assimilation rate. Chakhchar et al. (2017a) reported thus a close coordination of photosynthesis and stomatal conductance in four *A. spinosa* provenances under control and drought conditions which is consistent with other species adapted to growth in arid environments. The link between CO_2_ diffusive resistance and photosynthesis is also noted during the short-term variation of leaf-air VPD as tree argan stomata close when the demand for evapotranspiration increases (Chakhchar et al., 2017a).

Argan trees have developed adaptive mechanisms to tolerate drought stress through various biochemical and physiological processes including, among others, leaf water status, ionic homeostasis, osmoregulation, antioxidant system, secondary metabolism, carbohydrate metabolism, lipid metabolism (Chakhchar et al., 2015a, b,c, 2016, 2017b, 2019), leaf morphology (Díaz-Barradas et al., 2013; Zahidi et al., 2013c; Chakhchar et al., 2018b), chlorophyll fluorescence, photosynthetic carbon metabolism (Díaz-Barradas et al., 2010; Chakhchar et al., 2018b, c), and photosynthetic gas exchange (Chakhchar et al., 2017a; Table 1). These mechanisms assist in plant growth and tolerance to arid environments, characterized by high evaporation demand and low water availability (Chakhchar et al., 2017a). The argan tree also reveals a significant and rapid physiological and biochemical recovery in response to rehydration after severe drought stress (Chakhchar et al., 2016).

**TABLE 1:** Summary of some eco-physiological studies on drought adaptive mechanisms in *Argania spinosa.*

Adaptive mechanism	Adaptive traits	References
Water status	– Decrease of leaf water potential, stomatal conductance and relative water content. The stomata were highly sensitive to increased leaf to air vapor pressure deficit – Close correlation between epicuticular wax load (EWL) and residual transpiration rate (RTR) (increase of EWL and decrease of RTR)	El Aboudi et al., 1991; Díaz-Barradas et al., 2010, 2013, Chakhchar et al., 2015b, c
Ionic homeostasis and osmoregulation	– Increase in leaf concentrations of potassium (K^+^), calcium (Ca^2+^) and magnesium (Mg^2+^) – Increase of proline and soluble sugar content	Chakhchar et al., 2015b, 2017b
Antioxidant system	– Induction of enzymatic and non-enzymatic defense systems: – Increase in the activity of ascorbate-glutathione cycle enzymes: ascorbate peroxidase, monodehydro-ascorbate reductase, dehydroascorbate reductase, and glutathione reductase – Increase in the activity of catalase, superoxide dismutase, peroxidase, polyphenoloxidase, and glutathione S-transferase – Increase in the content of ascorbic acid, α-tocopherol, and reduced glutathione – Increase in the thiol protein content	Chakhchar et al., 2015a, c, 2017b, Lamaoui, 2015, Lamaoui et al., 2015, 2019
Secondary metabolism	– Induction of shikimate and phenylpropanoid pathways (stimulation of the activity of phenylalanine ammonium lyase, shikimate dehydrogenase, and cinnamate-4-hydroxylase enzymes) and increase of total polyphenols content	Chakhchar et al., 2015b
Carbohydrate metabolism	– Stimulation of carbohydrate enzymes: aldose 6-phosphate reductase, glucose-6-phosphate dehydrogenase, acid invertase, and sorbitol dehydrogenase – Accumulation of hexoses (glucose and fructose)	Chakhchar et al., 2018b, 2019
Pigments, photosynthetic carbon metabolism and gas exchange	– Decrease of leaf chlorophyll content and increase of leaf anthocyanin concentration – Maintain of the photosynthetic activity at low levels of foliar water content and co-ordination of photosynthesis, stomatal behavior, and metabolism – Positive relationship between the rate of respiration in the dark and Photosynthesis, indicative of coordination of metabolic and photosynthetic activity	Díaz-Barradas et al., 2010, Chakhchar et al., 2017a, 2018c

Such studies are expected to provide basic information about the ability of argan trees to adapt to drought stress while considering the magnitude of genetic variation in the adaptive traits. In fact, the process of adaptation to various environmental stresses is characterized by polygenicity and probable genetic heterogeneity. The methods of research in domestication programs should combine field ecophysiological studies and molecular genetic approaches, taking into account the heritability of each adaptive trait and the correlation between traits. The control of pollination and gene flow between *A. spinosa* ecotypes with good tolerant characteristics is expected to increase adaptation and natural regeneration. In addition to these scientific research approaches, it is necessary to opt for good practices in the fields in order to mitigate the environmental degradation that constrains argan forest growth, and thus influences oil quality and/or yield. The planting of argan tree orchards with rainwater harvesting and soil conservation capabilities appears to be an alternative to the rehabilitation of natural argan forest. The implementation of the argan tree orchards on private land from conventional agriculture, and also the adoption of some soil and water conservation technologies and practices which take into account the leaching requirement to avoid salt accumulation in the rhizosphere, the minimum superficial tillage, the mulching, the organic amendment application, etc., will be a great asset for the development of the arganiculture sector.

## Ecological Socio-Economic Aspects

### Ecological Interests

The alternation of long dry periods and short rainy periods is one of the characteristics of southwestern Morocco, with prolonged drought periods contributing to degradation of the dominant but vulnerable ecosystems. Due to its perfect adaptation to the soil and climate in this region (Díaz-Barradas et al., 2010, 2013), the argan tree plays an irreplaceable ecological role, including in water conservation, climate change mitigation, and soil stabilization in its territory, which is threatened by such biophysical processes as advancements in desertification and erosion (Parish and Funnell, 1996; de Waroux and Lambin, 2012; Alba-Sánchez et al., 2015). Indeed, it protects the soil against wind erosion, which is a constant threat in sublittoral zones located in the open wind sector. Argan trees are also exposed to water erosion, especially in mountainous areas and watersheds. *A. spinosa* is also known to stabilize soils on the slopes of the mountains, due to its powerful roots. Moreover, the root system helps with the uptake of soil water from a depth between 4 and 8.5 m (Ain-Lhout et al., 2016). Using the geophysical technique of electrical resistivity imaging, Ain-Lhout et al. (2016) have reported that the soil moisture content changed substantially over time and was much lower in summer than in spring. During the experimental period ranging from April to July, the resistivity values ranged between 2 and 18 Ωm. The availability distribution of soil moisture is mostly affected by precipitation, evapotranspiration, and soil hydraulic properties.

Nonetheless, higher soil moisture remains available, mainly underneath the argan roots, and the microbial activities are more important especially with regard to nitrogen mineralization and phosphorus availability (Nouaim and Chaussod, 1994; Nouaim et al., 1994). The argan tree is also involved in the formation and enrichment of the soil by directly promoting and improving its fertility through dead leaves and roots and indirectly by favoring vegetation that can grow under its shelter. Thus, the argan tree provides shade (shade effect) and a microclimate (litter effect) that are favorable to the development of many living organisms (flora, fauna, and microflora), considerably increasing the biodiversity of these natural areas (Nouaim et al., 1991; Nouaim and Chaussod, 1994; Ahansal et al., 2008). The argan forest provides an adequate environment for the development of a farming system, including intercropping and co-culture with forage crops or aromatic plants. Thus, the conservation and regeneration of argan forest can improve soil conservation and fertility as well as increase carbon storage in soil and biomass.

### Socio-Economic Interests

The natural argan forest has a specific legislative status (Dahir of 4 March 1925 and specifications relating to the agrarian practices under the argan tree of 20 July 1983), which makes it a national forest (state property) whose usufruct rights are dedicated to the local populations in an extensive manner. In this arganeraie, more than 1.3 million people are involved in the exploitation of traditional agroforestry systems based on the argan tree (Chaussod et al., 2005); however, local communities have been abandoning these traditional usage rights, as a result of the argan oil boom and socioeconomic changes, by acquiring new knowledge and developing important skills related to modern management and forest conservation (Lybbert et al., 2002, 2010; Chaussod et al., 2005).

Since December 1998, the Moroccan argan tree has been part of the World Network of Biosphere Reserves supported by UNESCO. This Arganeraie Biosphere Reserve forms a cluster comprised of 18 core areas, 13 buffer zones, and 14 transition zones [Haut-Commissariat aux Eaux et Forêts et à la Lutte Contre la Désertification [HCEFLCD], 2021]. The argan forest offers multiple functions (e.g., the creation of a favorable microclimate for many fauna and flora, soil erosion protection, and desertification control) and uses for local populations, whose socioeconomic activities are strongly linked to the various products that it provides (e.g., oil, soap, shampoo, cosmetic creams, and livestock feed) (Lybbert et al., 2002, 2010). Thus, this species offers diversified economic opportunities, through various emerging sectors (e.g., argan oil, ecotourism, and local products). The argan tree is a multipurpose tree; each part or product of the tree (e.g., wood, leaves, fruits, and oil) is usable and can serve as a source of income or food for the user (M’Hirit et al., 1998). This relic tree provides excellent firewood, which is also used to make utensils and tools for family or agricultural use. It is part of a natural resource in food and feed, through its forage production and oil. Indeed, *A. spinosa* provides a very precious oil, edible in its virgin state and strongly requested due to its organoleptic characteristics and its nutritional properties. Moroccan argan oil is now the most expensive edible oil in the world (Lybbert et al., 2010). This oil is also used in cosmetic preparations and has been scientifically proven to have medicinal, pharmacological, and therapeutic potentialities (Charrouf and Guillaume, 2008, 2011; Lopez Saez and Alba-Sánchez, 2009; Rammal et al., 2009; El Monfalouti et al., 2010). Virgin argan oil contains about 20% saturated fatty acids and at least 80% unsaturated fatty acids, including 35% polyunsaturated fatty acids and 45% monounsaturated fatty acids (Ait Aabd et al., 2013). It is characterized by high levels of linoleic and oleic acids (mean content of 38 and 45%, respectively) and is also rich in polyphenols, carotenes, and tocopherols (γ-tocopherol is the major fraction of about 84–86%) which have an important antioxidant activity (Cayuela et al., 2008; Charrouf and Guillaume, 2008; Marfil et al., 2011).

Over the past two decades, the market for argan oil and other argan products has evolved dramatically, such that the main interest regarding the argan tree, in the eyes of the local population, is now its market value as a result of the “argan oil boom.” In fact, as a result of soaring prices, new aggressive behaviors with regard to the argan tree have arisen for some farmers, rights-holders, and beneficiaries. On the other hand, due to competition in the market, local cooperatives have been weakened by many factors (e.g., weak link and access to trees, commercialization and marketing problems, illiteracy, and lack of experience in management).

Argan trees ensure the subsistence of a large rural population in southwestern Morocco, limiting the rural exodus. Indeed, 19% of the revenues of the local population depend on this tree (Benchakroun, 1990 in Majourhat et al., 2007). In fact, the argan forest, which is the hinterland of the Moroccan tourist metropolis, can be a space for decongestion and spatial rebalancing, which is a response to other types of tourism demands such as ecotourism, cultural tourism, and mountain tourism (Aboutayeb, 2014).

The integration of an appropriate management system for the argan tree, taking advantage of the synergies resulting from the interaction between ecological conservation and socio-economic development is a priority for the success of the agro-fruit-forest model. The adaptive behavior of the argan tree in its territory, threatened by desertification, triggers the way for the future direction of research; that is, to develop desert crop projects. Thus, it is vital to scientifically identify and delineate the areas of potential desertification in southern Morocco in order to facilitate forest plantations by integrating the argan tree as green dams. However, the economic evolution of the argan sector has had other unintended negative consequences on socioeconomic and cultural heritage in the “arganeraie agro-ecosystem.” Indeed, at the local level, this economic function of the argan tree has begun to overshadow its important social, psycho-emotional, and cultural functions in this society, which adds to the further complexity of its valorization, considering also practices and stakeholders. Hence, there is a need to seriously consider co-management of the arganeraie agro-ecosystem and the structure of the argan oil market and its products with respect to the synergy and coordination between the concerned actors.

## Regeneration, Domestication, and Arganiculture Strategy

Despite the social, agricultural, and economic importance of the argan tree, the argan forest area decreased by 44.5% between 1970 and 2007 (de Waroux and Lambin, 2012) as a consequence of a longer decline since the 18th century (McGregor et al., 2009). Thus, current developments, both economic and social, can cause changes in management practices throughout the argan forest (Lybbert et al., 2002, 2010; Alados and El Aich, 2008; Genin and Simenel, 2011; Karmaoui, 2016; Genin et al., 2017). Argan forest sustainability is now threatened by overgrazing and overexploitation in mountainous areas, along with the intensive agriculture in lowland areas (Charrouf and Guillaume, 2009; El Wahidi et al., 2014; Genin et al., 2017). This biogeographical variation is a determining factor for management strategies and the exploitation of genetic argan tree resources. It can also be relevant for domestication and conservation programs, as well as for abiotic responses resulting from climate change (Leakey et al., 2012). Future research projects to determine the structure of the genetic variation of natural, managed, and cultivated argan trees are particularly important for the preservation of argan genetic diversity, as well as to select appropriate management strategies.

In addition to the biodiversity of this natural resource, the Moroccan argan forest also forms the locale of an important civilization (Lopez Saez and Alba-Sánchez, 2009). It typically constitutes rural and domestic forest, as it integrates production and conservation with social, political, and spiritual dimensions (Genin and Simenel, 2011). Consequently, preservation of the remaining argan trees and the rehabilitation of degraded areas are becoming an urgent necessity in the southwest of Morocco (Genin et al., 2017).

**FIGURE 3:**
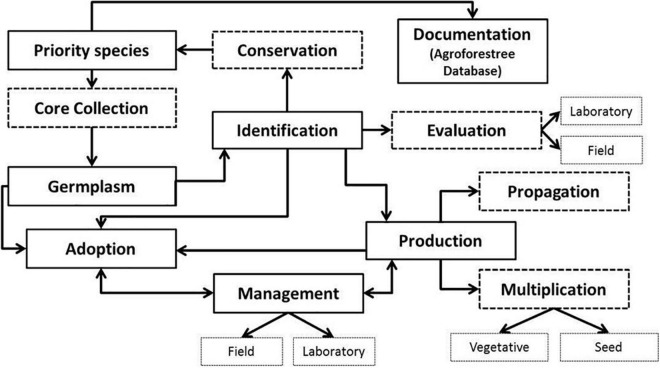
Schema indicating possible pathways for domestication of argan tree in Morocco.

The regeneration of *A. spinosa* could, therefore, be an effective tool to minimize the additional loss of the argan forest and prevent desertification (Genin et al., 2017). This species, with its international market, constitutes considerable potential for domestication programs, as for other indigenous African fruit and nut trees (Leakey et al., 2012). Projects focused on the establishment of arganiculture at a large scale have been implanted for the development of a modern horticultural industry in order to contribute to its domestication by adopting water efficient technologies coupled with a solar pumping system (Agence Nationale pour le Développement des Zones Oasiennes et de l’arganier [ANDZOA], 2020). The success of argan tree regeneration requires the selection of healthy plants with good genetic and agronomic qualities (Ait Aabd et al., 2011, 2012, 2015; Zahidi et al., 2014a; Chakhchar et al., 2017a), allowing for a controlled process (i.e., Tree Resource Domestication) of cultivation and environmental monitoring as well as the selection of cultivars to ensure consistent quality (Figure 3). The support of the local population in the argan Biosphere Reserve, through the planting of argan orchards and the promotion of sustainable arganiculture, will help to foster sustainable argan development, thus improving the living conditions of populations by increasing their incomes, building resilience, and supporting climate mitigation (Charrouf and Guillaume, 2009; Genin and Simenel, 2011; Genin et al., 2017).

Substantial knowledge on the ecological and agronomic properties and performances of argan tree such as species’ genetics, growth conditions, yield potential, input responsiveness, etc., is important to facilitate and guide an efficient and robust path toward its domestication. Nonetheless, the management of the arganiculture might hold considerable risks of unsustainable practices (e.g., land right and acquisition, adherence of the population, lack or insufficient irrigation of argan seedlings, and deterioration of biodiversity, especially relict or/and endemic species). So, the right strategy to achieve the agro-fruit-forest program consists in the development of diversified and environmentally friendly land use systems, which adopt adequate measures for the protection, and preservation of the argan tree. Overall, this program will provide a sustainable pathway for rural development, leading to enhanced livelihoods and greater environmental benefits by delivering multifunctional agriculture.

## Conclusion

The domestication of the argan tree will open up considerable economic opportunities for Morocco, as the demand for quality argan oil is expected to grow in the future. These procedures and measures also contribute to relieving anthropic pressure on the natural argan forest, improving the livelihoods of the local population by moving from a model of fruit collection from natural forest toward the horticultural industry and with sustainable forest co-management. It is expected that the arganiculture program will enable conversion of this indigenous tree into a new crop as well as provide better access to germplasms, technology, and finances for the mutual benefit of all involved parties. In addition, the marketable products of argan trees are expected to become key agricultural products, such that the rights of local farmers will be protected instead of these products being considered common-property forest resources.

## Author Contributions

AC and ML designed, wrote, and edited the manuscript. IB, YE, AF-M, and CE critically reviewed the manuscript. All authors read and approved the final manuscript.

## Conflict of Interest

The authors declare that the research was conducted in the absence of any commercial or financial relationships that could be construed as a potential conflict of interest.

## Publisher’s Note

All claims expressed in this article are solely those of the authors and do not necessarily represent those of their affiliated organizations, or those of the publisher, the editors and the reviewers. Any product that may be evaluated in this article, or claim that may be made by its manufacturer, is not guaranteed or endorsed by the publisher.
